# Unsaturated fatty acids as high-affinity ligands of the C-terminal Per-ARNT-Sim domain from the Hypoxia-inducible factor 3α

**DOI:** 10.1038/srep12698

**Published:** 2015-08-03

**Authors:** Angela M. Fala, Juliana F. Oliveira, Douglas Adamoski, Juliana A. Aricetti, Marilia M. Dias, Marcio V. B. Dias, Maurício L. Sforça, Paulo S. Lopes-de-Oliveira, Silvana A. Rocco, Camila Caldana, Sandra M. G. Dias, Andre L. B. Ambrosio

**Affiliations:** 1 Laboratório Nacional de Biociências, Centro Nacional de Pesquisa em Energia e Materiais, Campinas, SP, Brazil, 13083-100; 2 Laboratório Nacional de Ciência e Tecnologia do Bioetanol, Centro Nacional de Pesquisa em Energia e Materiais,, Campinas, SP, Brazil, 13083-100; 3 Max Plack-partner group at the Laboratório Nacional de Ciência e Tecnologia do Bioetanol

## Abstract

Hypoxia-inducible transcription factors (HIF) form heterodimeric complexes that mediate cell responses to hypoxia. The oxygen-dependent stability and activity of the HIF-α subunits is traditionally associated to post-translational modifications such as hydroxylation, acetylation, ubiquitination, and phosphorylation. Here we report novel evidence showing that unsaturated fatty acids are naturally occurring, non-covalent structural ligands of HIF-3α, thus providing the initial framework for exploring its exceptional role as a lipid sensor under hypoxia.

HIF, closely linked to disorders of the circulatory system and cancer progression^1^, consists of two multidomain subunits including the constitutively expressed HIF-1β and an oxygen-labile HIF-1α (or paralogs HIF-2α and -3α). The gene encoding for HIF-3α (*HIF3A*) is exclusively susceptible to post-transcriptional processing in humans, with six isoforms shown to exist at mRNA levels and three already confirmed as functional protein^2^,^3^. HIF-3α9, the longest (669 aa) and canonical isoform^3^, is a potent oxygen-labile transcription factor, tightly regulating the expression of a unique set of genes^4^. HIF-3α4 (363 aa), on the other hand, while insensitive to intracellular oxygen levels and incapable of transactivation, is a dominant-negative regulator of HIF-1α^5^. Due to the use of different transcription initiation sites and a combination of splicing events in *HIF3A*, the C-terminal Per-Arnt-Sim sensor domain (PASb) is the only common architectural feature among the HIF-3α isoforms^3^. The PASb domains in both HIF-α and -β subunits have a crucial role in forming active HIF heterodimers and recruiting co-regulators^6^,^7^.

PAS members are versatile sensory modules of stimuli such as oxygen tension, redox state and light intensity through binding to cofactors such as heme, flavin adenine dinucleotide and even divalent metal ions^8^. In the case of the HIF proteins, PAS domains-mediated protein-protein interactions have been shown independently of a cofactor and simply based on the presence of the partner PAS^9^,^10^. However, recently, the identification of a 290 Å^3^ polar cavity in HIF-2α PASb domain led to the proposition that endogenous ligands may exist^11^; this cavity was later exploited for the selection of artificial small molecules that disrupt the full-length HIF-2 heterodimer^10^,^11^,^12^.

Hereby, we present the crystal structure of the recombinant human HIF-3α PASb, or PASb-3α, at 1.15 Å maximum resolution (Table 1 and Supplementary Fig. 1). Although with a backbone virtually identical to that of the PASb domains of both HIF-1α^9^ and HIF-2α^10^ (Fig. 1a), PASb-3α has a unique extensive C-shaped hydrophobic cavity of 510 Å^3^ (Fig. 1b). We identified a 1-(11Z-octadecenoyl)-sn-glycerol molecule of bacterial nature buried in this cavity, as clearly suggested by the size and geometrical features of the electron density map (Fig. 1c, left panel). As such, the aliphatic tail of the fatty acid extended to eighteen carbons in length (maximum length limited by the cavity size), with a *cis* double bond between C11 and C12, interspersed by four gauche configurations (at positions C5, C7, C9 and C13, (Fig. 1c, right panel)). The ester group at the glycerol sn-1 position makes polar contact with the main chain carboxyl group of His339 and main chain amino group of Leu340. The sn-2 position is not substituted, also sharing a hydrogen bond with the Leu340 main chain amino group (Fig. 1d). Based solely on the electron density map, we could not tell whether the glycerol sn-3 position was substituted, due to absence of well-structured atoms beyond its remaining hydroxyl group. However, the presence of a well-coordinate sulfate ion (abundant in the crystallization solution), 6 Å apart from the glycerol sn-3 hydroxyl, suggested that PASb-3α could also bind to phospholipids (Fig. 1d).

Aiming at experimental confirmation of the identity of the proposed monoacylglycerol, the hydrophobic fraction was carefully isolated from pure protein samples in solution and analyzed by thin-layer chromatography (TLC). By applying two mobile phases distinct in polarity, as well as dual staining to allow for differential detection of neutral and phosphorous-containing lipids^13^, we surprisingly observed the dominant presence of two neutral lipids and two phospholipids as ligands of PASb-3α (Fig. 2a).

Accordingly, mass spectrometry analysis on the isolated hydrophobic fraction readily identified two differential peaks with m/z of 478.40 and 509.36 (Supplementary Fig. 2a,b), subsequently determined to be the lyso-phospholipids 1-(11Z-octadecenoyl)-2-hydroxy-sn-glycero-3-phosphoethanolamine, or 18:1-LPE, and 1-(11Z-octadecenoyl)-2-hydroxy-sn-glycero-3-phospho-(1´-sn-glycerol), or 18:1-LPG, respectively (Fig. 2a), in accordance with the TLC analysis. Experimental limitations related to the ionization of the neutral lipids kept us from identifying the precise nature of such.

Collectively, all complex lipids identified as PASb-3α ligands include the 18-carbon unsaturated 11Z-octadecenoic acid (or cis-vaccenic acid; 18:1, cis-11) and an unmodified sn-2 glycerol position as common structural denominators. Given the heterologous nature of the expression system used for human PASb-3α production, it is key to mention that cis-vaccenic is the sole 18-carbon unsaturated fatty acid found in *E. coli*^14^.

Next, in order to quantitatively check whether the degree of unsaturation influenced the binding of 18-carbon fatty acids to PASb-3α, we performed a fluorescence-based assay in the presence of the hydrophobic probe 1,8-ANS, upon the titration of fatty acids to the delipidated protein. Intriguingly, upon fatty acid titration, no clear dose-response curve was observed for stearic acid (18:0), while sigmoid curves could be fitted in response to both oleic (18:1; cis-9) and linoleic acids (18:2; cis-9,12) (Fig. 2b). An apparent K_d_ of 39 nm for the monounsaturated oleic acid was determined, which improved 50-fold (up to 0.8 nM) for the polyunsaturated linoleic acid. Therefore, in addition to showing that 18-carbon fatty acids that occur in human cells, such as oleic and linoleic acids, are high-affinity ligands of PASb-3α, we also establish that unsaturation is a required feature for fatty acid binding and the greater the degree of unsaturation, the stronger the interaction with the protein becomes.

The mechanism by which the lipid molecule enters the PASb-3α pocket is unclear. However, the outcomes of the binding assay above can be reasoned from the perspective of the free fatty acids, since the ligand conformations in the bound state are dependent on the frequency of such microstates as part of the unbound ensemble in vacuum (thus simulating lipid desolvation). Upon ensemble recapitulation, it is curious to observe that, while the putative bound conformation of stearic acid has the lowest conformational enthalpy inside the pocket (−3 kcal/mol), such a conformation has very low probability of happening upon desolvation (Fig. 2c). On the other hand, the bound conformations of oleic and linoleic acids, although with higher enthalpies (4 and 37 kcal/mol, respectively), are more frequent and easily achieved since the estimated ensemble entropies are significantly smaller than that of stearic acid (Fig. 2c).

NMR and fluorescence polarization data showed that purified, lipid-associated PASb-3α, has stronger association with HIF-1β PASb, albeit with slower binding kinetics, when compared to the well-characterized HIF-1α:HIF-1β PASb dimer (Supplementary Fig. 3a–e). In hopes of characterizing exactly how the absence of lipids could affect the heterodimerization of PASb-3α to HIF-1β PASb, we performed extensive attempts to remove the accompanying bacterial lipids from the purified protein by conventional hydrophobic interaction chromatography, reversible unfolding and detergents, all of which failed. Limited success was attained after treatment with the LIPIDEX-1000 matrix allowing, however, the recovery of only minor amounts of protein^15^. This observation may point to the role of the lipids as a structural cofactors for PASb-3α, which is not without precedents, since some ligand-free PAS have already been shown to be predominantly unstructured in solution and prone to precipitation^16^,^17^. A fitting example is the association between the ligand-free PASb domain of the Aryl Hydrocarbon Receptor (AHR), with a predicted similarly large hydrophobic cavity (500 Å^3^) used for ligand binding, and the chaperone *Heat shock protein 90* (Hsp90). The presence of compounds known to activate the AHR response pathway displaces Hsp90 from this complex, suggesting that these chemicals stabilize a folded form of the PASb domain^18^. Therefore, we suggest that the binding of lipids to PASb is mandatory for the proper folding and stability of HIF-3α, inducing its translocation to the nucleus and subsequent activation of HIF function. This hypothesis is strongly supported by the direct observation that HIF-3α indeed co-precipitates with HSP90 in cell models, likely via its PASb domain^19^. However, the dependence of this interaction on the presence of lipids has yet to be tested.

Curiously, the splicing event that gives rise to the HIF-3α4 isoform completely changes the amino acid sequence for the second half of its PASb domain^3^ (Supplementary Fig. 4a). *Ab initio* homology modelling of PASb-3α4 strongly suggest that amino acid changes relative to PASb-3α (Supplementary Fig. 4a), result in substantial deformation of the topography of the hydrophobic cavity as a consequence of the presence of longer, bulkier and polar side chains inside the cavity (Supplementary Fig. 4b). These observations strongly suggest that, out of all HIF-3α isoforms, the splicing variant HIF-3α4 - which is uniquely insensitive to cellular oxygen levels^5^ - may as well be exclusively insensitive to lipids. Of note, HIF-3α4 is a negative regulator of HIF-1^5^. Extensive attempts to produce PASb-3α4 domain in bacterial system only resulted in insoluble protein products, hindering the testing of our hypothesis.

With all the possible scenarios described above several outstanding questions arise. These include the identification of which lipids may in fact regulate HIF-3α function, for instance in cancer (Supplementary Figs. 5a–b and 6a–d) or obesity^20^, under what exact circumstances lipids must be sensed and what are the possible outcomes of lipid binding. However, by defining PASb-3α as a lipid-binding domain, our findings lay the groundwork to explore the role of HIF-3α as a link coupling lipid sensing under hypoxia, thus adding an unanticipated layer of complexity to the already intricate mechanism for HIF-mediated gene expression. Finally, similarly to HIF-2α, the description of an exclusive hydrophobic pocket yield unique implications for the future development of small molecule-oriented therapeutics targeting specifically HIF-3α.

Collectively, our structural and biochemical observations also carry implications for the overall molecular aspects of the PAS fold. For the first time, we document that a eukaryotic PAS domain may have lipids as high-affinity cofactors, thus introducing a new class of PAS ligands and expanding its already vast functional portfolio^8^.

## Methods

### Cloning

PASb-1α (Asp238-His348, UniProt Q16665), PASb-1β (Pro349-Ser467, UniProt P27540) and PASb-3α (Gly237-Thr345, UniProt Q9Y2N7-1) coding sequences were amplified by Polimerase Chain Reaction from a cDNA library from the breast cancer cell line SKBR3. PASb-1α and PASb-3α constructs were cloned into the pET28a plasmid (Novagen) using the *NdeI* and *XhoI* restriction sites. PASb-1β was cloned into a modified version of pETSUMO vector (Invitrogen) using *BamHI* and *HindIII* restriction sites. The following pairs of oligonucleotides were used: PASb-1α – Forward: 5′-AGTCTCATATGGATAGCAAGACTTTCCTCAGTCGA-3′ and Reverse: 5′-CGTACCTCGAGTTAGTGCTGAATAATACCACTCAC-3′; PASb-1β – Forward: 5′-TTGGTGGATCCCCCAACTGTACAGACATGAGTAAT-3′ and Reverse: 5′-GCCGCAAGCTTTTAAGAGTTCTTCACATTGGTGTT-3′; PASb-3α – Forward: 5′-AGTCTCATATGGGCCGAGGGGCCTTCCTCAGCCGC-3′ and Reverse: 5′-CGTACCTCGAGTTAGGTCTCTTCCACCTGGCTGAT-3′.

### Expression and purification of recombinant proteins

The following expression and purification protocol was applied to the three Pasb constructs, transformed into *Escherichia coli* Rosetta-2 thermocompent cells (Merck). Overnight cultures, grown in LB medium supplemented by 50 μg.ml^−1^ kanamycin and 50 μg.ml^−1^ cloramphenicol, were inoculated in a ratio of 1:100 in 5 liter cultures supplemented with the same antibiotics and left shaking at 200 rpm for 5 hours at 37 ^o^C. The cultures were then down-tempered to 18 °C for 1 hour before induction with 0.2 mM IPTG (isopropyl β-D-1-thiogalactopyranoside) for additional 16 hours at 18 °C. Cells were harvested by centrifugation (5,000 × g) for 10 min at 4 °C and resuspended in buffer containing 50 mM Tris-HCl pH 7.5, 150 mM NaCl, 10% glycerol, 2 mM BME (β-mercaptoethanol) and 1 mM phenylmethylsulfonyl fluoride. Cell lysis was performed chemically by incubation with hen egg-white lysozyme, DNAse I and deoxycholate (all three reagents from Sigma-Aldrich) for about 1 hour, incubated on ice. The soluble fractions were separated from the debris by high speed centrifugation (23,000 × g) and subsequently loaded, by gravity, on Co^2 + ^-charged resin TALON (Clontech), previously equilibrated with the running buffer 50 mM Tris-HCl pH 7.5, 30 mM NaCl and 2 mM BME. The constructs, bearing a 6xHis-tag fused to their N-terminus, were eluted stepwise using running buffer to which up to 500 mM imidazole (v/v) was added. The tags were removed by overnight digestion with either bovine thrombin (Sigma-Aldrich) or ULP-1 protease and the samples loaded into a MonoQ 5/50 GL anion exchange chromatography column (GE Healthcare). Elution was done by performing a linear gradient with a buffer containing 1 M NaCl, 30 mM Tris-HCl pH 7.5 and 2 mM BME. The fractions containing the protein of interest were loaded into a HiLoad 16/600 Superdex 75 size-exclusion column (GE Healthcare), equilibrated with 30 mM Tris-HCl, pH 7.5, 150 mM NaCl and 0.5 mM TCEP (tris(2-carboxyethyl)phosphine). Protein concentrations were determined by UV 280 nm using calculated extinction coefficients.

### Crystallization

Size-exclusion purified PASb-3α was concentrated down with an Amicon ultrafiltration device (10 KDa cutoff; Millipore) to a final concentration of approximately 300 μM. Crystallization experiments were performed at 291 K using the conventional sitting drop vapor diffusion technique. Drops were made by mixing equal parts of protein and well solution, containing 3% PEG 400, 1.9 M Amonium Sulphate, 0.1 M Sodium Acetate pH 5.7. Small bar-shaped crystals started growing two days after the setting up of the crystallization drops, reaching about 50 μM in longest dimension on average usually after 5 days. Before data collection at cryogenic temperature (100 K), harvested crystals were cryoprotected with 10% ethylene glycol added to the mother liquor (v/v).

### X-ray crystallography

X-ray diffraction data sets were obtained at the P13 beamline at PETRA III, Hamburg, Germany. Data was processed using Mosflm^21^ and merged and scaled with Aimless^22^. The first set of phases was obtained by the molecular replacement as implemented in the program Phaser^23^, using the PASb domain of HIF-1α (PDB ID 4h6j^9^) as a search model. Positional and anisotropic B-factor refinement cycles were carried out with Phenix^24^. Manual building of the extra portions and real space refinement, including Fourier electron density map inspection, were performed with Coot^25^. Solvent water molecules, treated as oxygen atoms, were added using the appropriate Coot routine. The survey for cavities was done using KVFinder^26^. During model refinement, the inspection of Fourier difference maps indicated the presence of very strong non-protein electron densities (over 3σ in height in the F_obs_-F_calc_ Fourier maps) that were interpreted - due to their sizes and shapes and in respect with the crystallization condition – as two 1-11Z-vaccenoyl-sn-glycerol molecules, five PEG 400 molecules and two sulfate ions, all subsequently refined as such. Two additional strong electron density distributions also resembling PEG400 molecules were found on the vicinities of Tyr264 in chain A and Trp315 in chain B. The refinement of such molecules, however, did not converge after many modeling configurations and the densities were then left as unexplained. The overall stereochemical quality of the final models and the agreements between them and experimental data were assessed by the program Molprobit^27^ and the appropriate Phaser and Coot routines.

### Thin-layer chromatography

Chromatographic separations were carried out on 4 × 2 cm TLC Silica gel 60 F_254_ aluminum plates (Merck). The complex lipid mixture was applied on the edge of the plates (at the sample spotting line) using a glass capillary. The samples were allowed to run for 4 cm in a 8 × 4 cm glass chamber, previously saturated with the mobile phase. Two distinct mobile phases were used - cyclohexane:ethyl acetate (4:1) and chloroform:methanol:water (75:25:2.5) - which respectively allow for the separation of neutral lipids and the separation of phospholipids by head group polarity. The plates were then left exposed to air to allow for evaporation of the mobile phase residual. For differential detection of neutral lipids and phosphorous-containing lipids, iodine vapor and a modified Dittmer–Lester reagent^13^ were employed for dual staining. The plates were scanned immediately after drying.

### Mass spectrometry

Lipids were extracted from 400 μL of purified protein (~1 mg.ml^−1^) using 1 mL of a precooled (−15 °C) mixture of MTBE: methanol: water 3:1:1 (v/v/v) as previously described^28^,^29^. Five hundred microliters of the upper phase, containing the lipids, were transferred to a fresh 1.5 ml Eppendorf tube and dried down in a speed-vac. The extracts that were reconstituted in acetonitrile: isopropanol 7:3 (v/v) (final concentration 25 μg.ml^−1^) were gradient-eluted on an UPLC-system (Accela, Thermo) using a reverse phase C8 column ACQUITY UPLC BEH 1.7 μm, 2.1 × 150 mm (Waters, 186003377). The gradient of separation was done at 400 μL.min^−1^ using (A) 0.1% acetic acid in water and (B) 0.1% acetic acid in methanol (60% B during 4 min, 60% B to 80% B in 4 min, 80–99% B in 0.5 min, 99% B for 3.5 min). The samples were measured in negative ion mode and mass spectra were acquired using an LTQXL mass spectrometer (MS) (ThermoFisher Corporation) with electrospray ionization (ESI). The MS interface capillary was maintained at 325 °C, with a sheath gas flow of 45 (arbitrary units) and auxiliary gas flow of 2 (arbitrary units) for negative injections. The spray voltage ion injection was 4 kV, and the instrument scanned 350–600 m/z. MS/MS normalized collision energy was set to 41, activation Q 0.25, activation time 30 ms, and a 2 m/z isolation window. MS/MS scans were collected using dynamic exclusion with an exclusion time of 30 s. Obtained raw-chromatograms were further processed using Excalibur software version 2.10 (Thermo Fisher). The MS/MS scores are based on a comparison of the ions present in the experimental spectrum to the ions present in the standard library spectrum from the Lipid Mass Database^30^,^31^.

### Fluorescence polarization

Purified PASb-1β was labeled with the FITC fluorophore (fluorescein isothiocyanate, Thermo Scientific) by mixing 1 mg of protein with 20-fold molar excess of FITC in 50 mM borate buffer pH 8.5 for 1 hour at room temperature on the dark. The excess FITC was removed by overnight dialysis at 4 °C with 30 mM Hepes pH 7.5, 150 mM NaCl and 0.5 mM TCEP. Unlabeled PASb-1α and PASb-3α, initially at a concentration of approximately 1 mM, were serially diluted 1:2 nineteen consecutive times into buffer 30 mM Hepes pH 7.5, 150 mM NaCl and 0.5 mM TCEP. Then, 18.75 μL from each protein dilution was mixed with 6.25 μL of PASb-1β labeled with FITC at 80 nM (final concentration of labeled protein was fixed at 20 nM). A final reaction volume of 25 μL was pipetted into a 384-well black-walled microplate (Greiner Bio-one), in triplicate. Protein:protein complex samples were incubated at room temperature in the dark and fluorescence polarization (FP) was measured in a CLARIOstar microplate reader (BMG Labtech) with excitation and emission wavelengths of 480 nm and 520 nm, respectively. Experimental values were output as mean ± standard deviation and logistic sigmoidal curves fitted to determine dissociation constants (K_d_) for each protein:protein complex interaction.

### Lipid binding assay

Removal of bacterial lipids from purified PASb-3α was reached by protein incubation (1.7 mL at 10 uM) with 100 mg of LIPIDEX 1000 (Sigma Aldrich) for 1 h at 37 °C in buffer containing 30 mM Tris pH 7.5, 150 mM NaCl and 0.5 mM TCEP, for three to five consecutive times. After delipidation the protein was incubated with different fatty acids for binding assay using the fluorescent probe 8-Anilino-1-naphthalenesulfonic acid (1,8-ANS). The final protein concentration used in the measurements was around 1 μM, with 10 μM of ANS. The fatty acid solutions, at initial concentration of 33 μM in pure ethanol, were diluted in ethanol by serially diluted 1:3 twelve consecutive times. The concentration of ethanol in the final reaction never exceeded 1%. The fluorescence measurements were recorded on a EnSpire multimode plate reader (Perkin Elmer) using a 96-well black-walled plate (Perkin Elmer), with excitation wavelength of 380 nm and emission wavelength range of 400–600 nm, at 25 °C.

### Isotopic labeling of PASb-1β

To prepare ^15^N- and ^13^C/^15^N-labelled PASb-1β, *Escherichia coli* Rosetta II cells were allowed to grow in a modified M9 minimal medium^32^ supplemented with 1 g.L^−1 15^NH_4_Cl (Cambridge Isotope Laboratories) combined with 4 g.L^−1^ D(+)-glucose (Sigma) to obtain only ^15^N-labelled proteins or with [^13^C6]-D(+)-glucose (Cambridge Isotope Laboratories) to obtain double-labeled proteins. Expression and purification of labeled PASb-1β was performed using the same procedure as described for the unlabeled protein.

### NMR spectroscopy

^15^N- and ^13^C/^15^N-labeled PASb-1β was produced and purified in a final buffer containing 30 mM Na_2_HPO_4_-NaH_2_PO_4_ pH 7.2, 70 mM NaCl, 0.5 mM TCEP and 10% D_2_O. NMR experiments were performed using an Agilent Inova spectrometer, operating at a ^1^H Larmor frequency of 599.887 MHz and temperature of 298 K. The spectrometer is equipped with a triple resonance cryogenic probe and a Z pulse-field gradient unit. The spectra were processed with NMRPipe/NMRDraw^33^ and analyzed with NMRView^34^. The ^15^NHSQC spectra of PASb-1β was collected and analyzed based on a previously deposited set of assigned chemical shifts into a public database at BioMagResBank (BMRB Entry 6597) and confirmed using the three-dimensional experiments HNCACB, CBCA(CO)NH, HNCO, and HN(CA)CO^35^,^36^,^37^,^38^. PASb-1α and PASb-3α, both initially at 900 μM, were titrated in ^15^N-labeled PASb-1β (200 μM), followed by the recording of the two-dimensional ^15^NHSQC spectra. Binding of proteins was characterized by changes in ^15^NHSQC signals, intensities and chemical shift values, as a function of the concentration of unlabeled protein. The chemical shift perturbation of the ^15^N-HSQC spectra was normalized according to the equation Δδ(^15^N + ^1^H) = [(Δδ^15^N/10)^2^ + (Δδ^1^H)^2^]^1/2^, in ppm units^39^ and the relative peak intensity changes calculated using the equation (I − I_0_)/I_0_, where I is the peak intensity measured in the presence of the maximum amount of unlabeled protein and I_0_ is the peak intensity measured in the free labeled protein.

### Molecular Modelling of PASb-3α4

*Ab initio* homology modelling of PASb domain of HIF-3α4, or PASb-3α4 (Gly237-Pro351, Uniprot Q9Y2N7-4) was done using methodology implemented by I-TASSER^40^. The top output model was used for comparison against the crystallographic model of lipid-bound PASb-3α.

### Tissue array

Human normal and tumor tissue arrays containing 78 normal tissue spots (FDA805-1, U.S. Biomax) or 18 normal tissues and 54 tumors (FDA805-2, U.S. Biomax) from multiple organs were subjected to immunohystochemical analysis with Anti-HIF3 alpha antibody (ab10134, Abcam). The tissue array slides were deparaffinized by two xylene washes (10 min each) and then rehydrated in ethanol series. After washing with water, the slides were microwaved for 10 rounds of 1 min in 10 mM sodium citrate buffer (pH 6.0) and allowed to cool to room temperature to retrieve antigens. Endogenous peroxidase activity was extinguished after 30 min incubation in 0.3% H_2_O_2_ in PBS. The slides were then blocked with Avidin and Biotin blocking solutions (Avidin/Biotin Blocking Kit; Vector Labs) and 1% goat serum in PBS (Vectastain Elite ABC Kit, Vector Labs). The tissue arrays were incubated overnight with the primary antibody (1:100 dilution in 1X PBS, 0.2% BSA, 0.02% Triton X-100, 10 mM Glycine), washed in same buffer, incubated with biotinylated secondary antibody 1:200 and treated for 30 min with the Elite ABC reagent (Universal R.T.U. Vectastain Elite ABC Kit; Vector Labs). After washing with PBS, the slides were processed with chromogen solution (ImmPACT DAB Substrate; Vector Labs). The slides were then dehydrated by ethanol series and mounted using Entellan New (Merck). The resulting staining obtained were captured using Operetta (PerkinElmer) 2X magnification objective, stitched by Harmony software (PerkinElmer) and quantified using ImageJ software. To allow comparison between slides, all data was normalized first to Skin Malignant melanoma spot (present in both slides). A second normalization was done to the highest expression in each specific tissue type. Samples were selected based on n ≥2 and the simultaneous presence of both normal and cancer representatives. Statistical significance was tested using Two-sample t-test, considering p < 0.1.

### TCGA data analysis

All the gene-expression data derived from RNA-seq were downloaded from The Cancer Genome Atlas data portal (http://tcga-data.nci.nih.gov/tcga/dataAccessMatrix.htm). For the evaluation of HIF-3α9 expression levels across tumor types, the normalized RSEM count from uc002peh.2 were used. For tumors with mean expression above 5, the 2-component Gaussian mixture distribution model with fixed variance for definition of expressing and non-expressing patients^41^,^42^ was implemented using mclust^43^ package (R statistical software^44^) and fitted independently for each tumor dataset using the uc002peh.2 expression levels. Patients were categorized in each class and gene differential expression analysis was performed using EBSeq^45^ package (R statistical software) using as input raw RSEM values and performing a 75h quantile normalization^46^. Genes up- (>1) and down- regulated (<1) with posterior probability of being differentially expressed above 0.95 were used as input for KOBAS2.0^47^ pathway enrichment analysis for REACTOME database. The Venn plot were made with VennDiagram^48^ package (R statistical software).

### Canonical ensemble simulation of lipid in vacuum

A Metropolis-Monte Carlo simulation was performed to recapitulate a canonical ensemble for the free stearic, oleic and linoleic acids. One-million-steps simulation, as implemented in Python, was performed, with temperature set to 300 K. The associated energies of randomly generated microstates was calculated using YASARA’s function Energy(“All”)^49^ and the AMBER03 force field^50^ for the parametrization of the lipid molecules. Probability of micro-states P(s) were calculated by discretization of Boltzmann distributions using hist(Energy)$density function of the statistical software R^44^. Entropy was estimated with S(lipid,vacuum) = P(s).log(P(s)).

## Additional Information

**How to cite this article**: Fala, A.M. *et al*. Unsaturated fatty acids as high-affinity ligands of the C-terminal Per-ARNT-Sim domain from the Hypoxia-inducible factor 3α. *Sci. Rep*. **5**, 12698; doi: 10.1038/srep12698 (2015).

## Supplementary Material

Supplementary Information

## Figures and Tables

**Figure 1:**
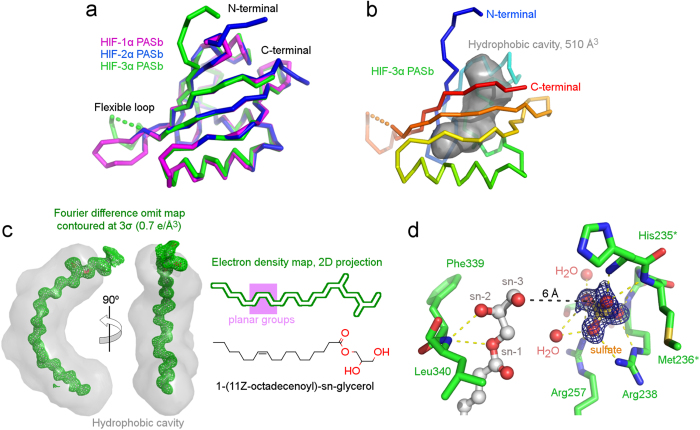
Structure of lipid-bound PASb-3α. **(a)** High structural identity between the main chain tracing of PASb-3α and the PASb domains of both HIF-1α and HIF-2α (similar core r.m.s.d. of 0.6 Å). **(b)** However, a 510 Å^3^ hydrophobic cavity was exclusively identified in the core of PASb-3α (delimited by gray surface), and (**c**) found to enclose a monoacylglycerol molecule. (**d**) Extensive network of polar contacts (yellow dashed lines) shared by specific residues in PASb-3α with the lipid and a neighbor sulfate ion, suggesting that minor rearrangements in the protein may allow for binding of phospholipids.

**Figure 2:**
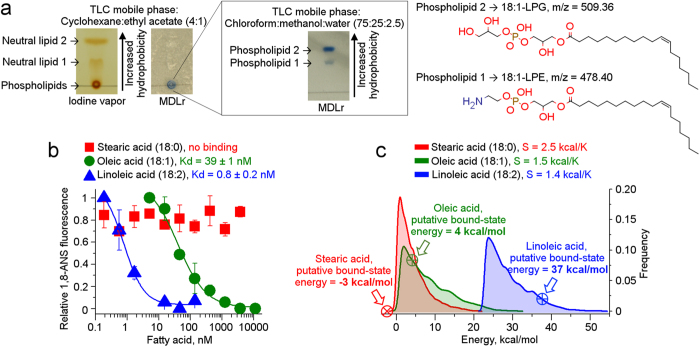
Characterization of the lipids bound to PASb-3α. (**a**) Two neutral lipids (yellow bands on the iodine vapor stained plate) and two phosphorous-containing lipids (blue bands over white background) were carried over by the purified protein samples. The two phospholipids were identified as 18:1-lysophospholipds. (**b**) Free oleic and linoleic acids bind with low-nanomolar affinity to delipidated PASb-3α, suggesting that unsaturation is a mandatory feature for the partner fatty acid and, as unsaturation accumulates, the higher the affinity for the protein. (**c**) Boltzmann distributions representing the canonical ensembles of fatty acid desolvation. The estimated entropies decrease as the degree of unsaturation increase. Crossed-circles over the curves point out the frequency of putative bound-state enthalpy into the distribution.

**Table 1:** X-ray crystallography data collection parameters and structure refinement statistics.

The C-terminal Per-Arnt-Sim (PASb) domain of human HIF-3α9 bound to 18:1-1-monoacylglycerol, PDB ID 4wn5			
Data Collection		Model Refinement	
Beamline	P13 at PETRA III	Resolution range (Å)	28.5–1.15
Wavelength (Å)	0.9770	Reflections (cross-validation)	68777 (5952)
Space group	P2_1_22_1_	R_factor_/R_free_ (%)	12.2/14.1
Cell parameters a, b, c (Å)	52.7, 53.9, 67.8	Average B-factor (Å^2^)	
Resolution range (Å)	28.5 – 1.15 (1.17 – 1.15)	main chain (no. of atoms)	8.8 (870)
Unique reflections	68827 (3381)	side chain (no. of atoms)	13.2 (812)
Multiplicity	9.8 (9.1)	ligands (no. of atoms)	19.9 (155)
R_pim_ (%)	1.9 (5.2)	solvent (no. of atoms)	28.0 (295)
CC (½)	0.998 (0.987)		
Completeness (%)	99.3 (99.4)	R.m.s.d. from standard geometry	
<I/σ(I)>	22.8 (11.5)	bond length (Å)	0.01
Average mosaicity (^o^)	0.5	bond angles (°)	1.36
B-factor Wilson Plot (Å^2^)	6.7		
Monomers/AU	2	Ramachandran plot,	
Solvent content (%)	36.1	favored (%)	100
Matthews coeff. (A^3^/Da)	1.92		

Data for outer shell are shown in parentheses.
