# Supplementary material for: Unsaturated fatty acids as high-affinity ligands of the C-terminal Per-ARNT-Sim domain from the Hypoxia-inducible factor 3α
Source: Sci Rep. 2015 Aug 3;5:12698. doi: 10.1038/srep12698 (PMC4522956; doi:10.1038/srep12698)
Supplement: Supplementary Information [file srep12698-s1.pdf]

SUPPLEMENTARY INFORMATION

Unsaturated fatty acids as high-affinity ligands of the C-terminal Per-ARNT-Sim domain from the Hypoxia-inducible factor 3α

Angela M Fala[1]*, Juliana F Oliveira[1]*, Douglas Adamoski[1], Juliana A Aricetti[2], Marilia M Dias[1], Marcio VB Dias[1], Maurício L Sforça[1], Paulo S Lopes-de-Oliveira[1], Silvana A Rocco[1], Camila Caldana[2,3], Sandra MG Dias[1]† and Andre LB Ambrosio[1]†

Laboratórios Nacionais de [1]Biociências e [2]Ciência e Tecnologia do Bioetanol, Centro Nacional de Pesquisa em Energia e Materiais, Campinas, SP, Brazil, 13083-100

[3]Max-Planck-partner group at the Laboratório Nacional de Ciência e Tecnologia do Bioetanol, Campinas, SP, Brazil, 13083-100

* These authors contributed equally to this work.

† These authors jointly supervised this work.

SUPPLEMENTARY FIGURES

Supplementary Figure 1.

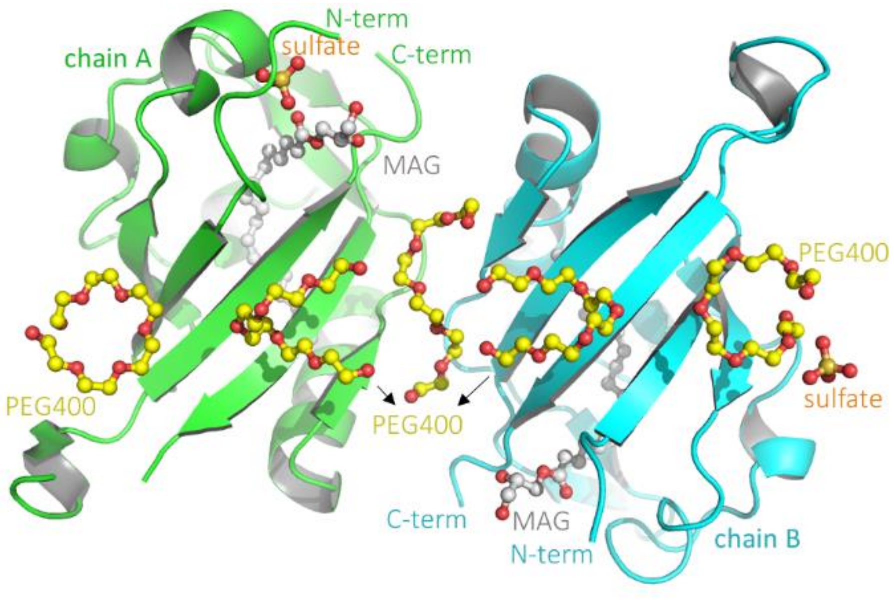

The crystal structure of human PASb-3α (Gly237-Thr345). The asymmetric unit of the PASb-3α orthorhombic crystal is composed by two protein monomers (green and cyan, in cartoon representation) bound to one 18:1-1-monoacylglyceride lipid molecule (MAG, white spheres) each, in addition to five PEG400 molecules (in yellow) and two sulfate ions. A PISA server[51] analysis suggested there to be no biological significance to the shared interface. One PEG400 molecule, present in abundance in the crystallization condition, mediates the monomer contacts in the asymmetric unit, while four additional PEG400 molecules mediate contacts with symmetry-related mates. The PASb-3α fold is characterized by one long and three short consecutive α-helices flanking a five-stranded anti-parallel β-sheet. Figure drawn by ALBA.

Supplementary Figure 2.

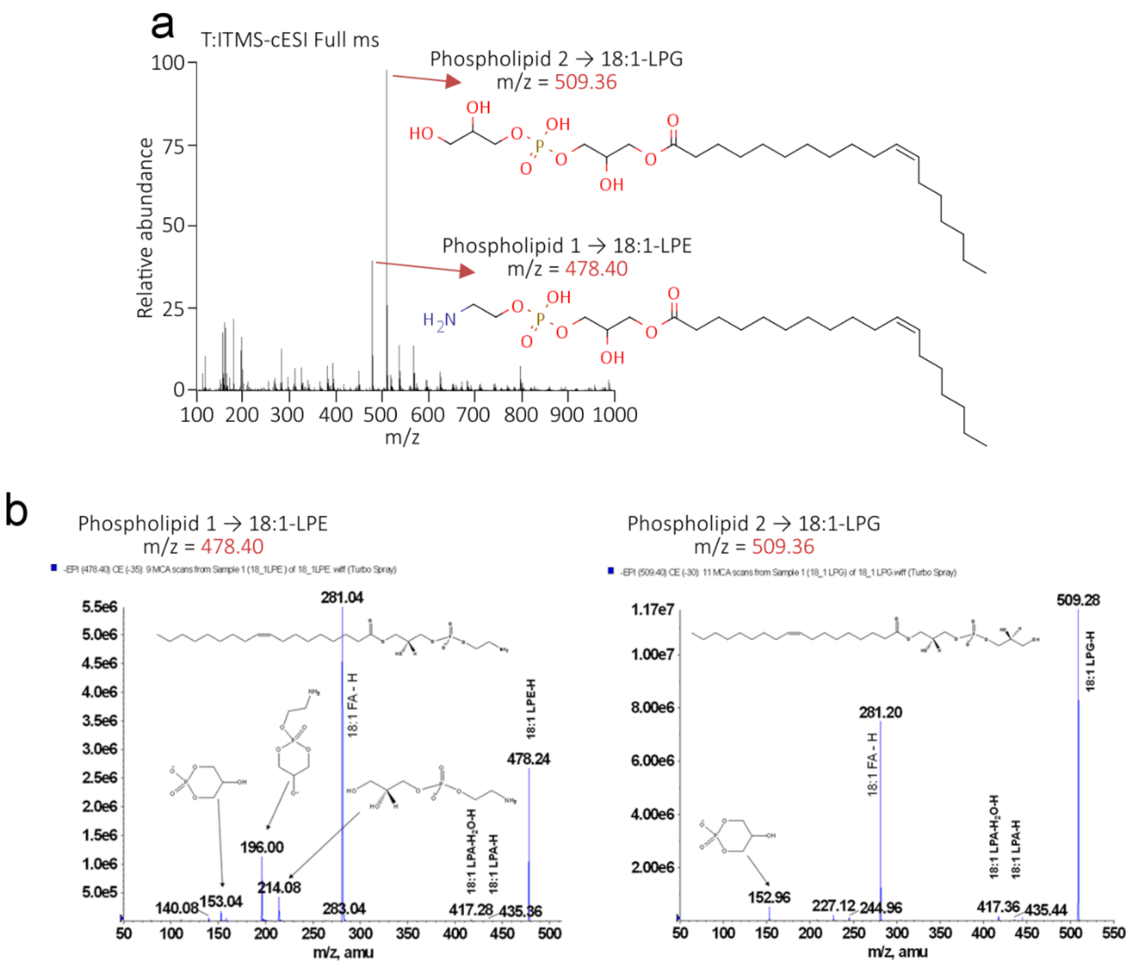

Identification of the bound bacterial lipids by mass spectrometry. Aiming at the precise description of such lipids, we performed mass spectrometry analysis on independent protein batches. **(a)** Two differential peaks could be immediately identified with m/z of 478.40 and 509.36. **(b)** Fragmentation analysis and comparison with Lipid Maps database[30,31] led to the matching of the mass peaks with phosphatidylethanolamine and phosphatidylglycerol with aliphatic chains ranging of 18 carbons. Both glycerophospholipids were identified in negative modes and in the deprotonated form.

Supplementary Figure 3.

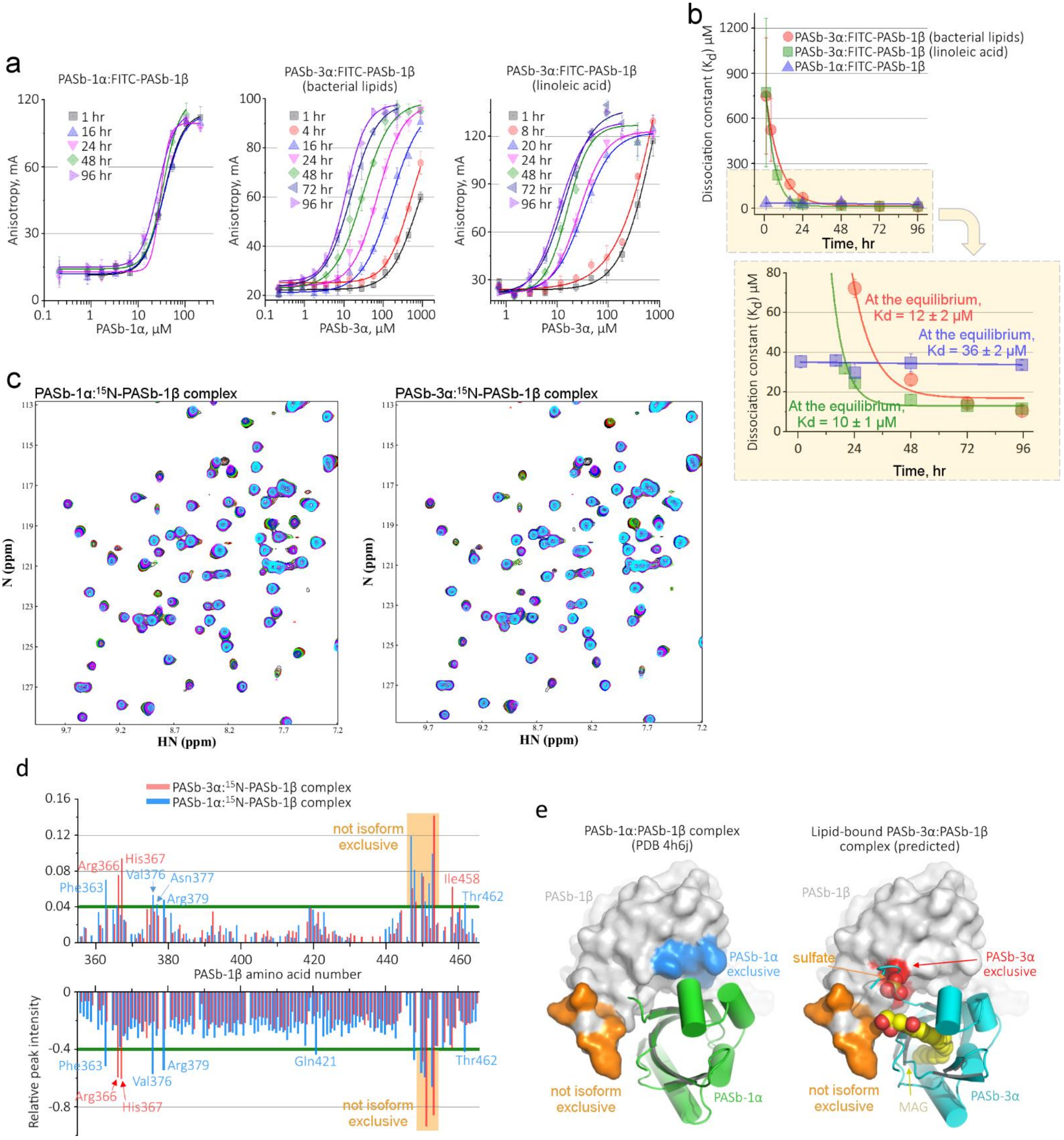

Lipids influence the PASb-3α:PASb-1β interaction. Since no other dimerization partner for the α subunits of HIF other than HIF-1β has ever been identified, we predict that HIF-3α acts via the canonical HIF α:β complex. In this context, we set out to probe the kinetics of dimer formation between PASb-3α and PASb-1β, using fluorescence polarization. **(a)**

FITC (fluorescein isothiocyanate)-labelled PASb-1β was incubated with increasing concentrations of either PASb-3α or PASb-1α (the later as a positive control) and the interaction was tracked as a function of time, for 96 hours. **(b)** Next, the extracted dissociation constants from the binding curves were plotted against the respective time points. While a stable dissociation constant (Kd) of 36 ± 2 µM was systematically obtained for the PASb-1α:PASb-1β complex (leftmost panel in **(a)** and blue triangles and solid line in **(b)**), when in the presence of the carried-over lipids, the PASb-3α:PASb-1β association improved as a function of the incubation time. During the first few hours the lack of well-defined binding curves resulted in the extraction of very weak and imprecise dissociation constants (middle panel in **(a)** and red circles in **(b)**). However, beyond 48 hours of incubation, the interaction further stabilized to a Kd of about 12.4 ± 2 µM. Taken together, the overall decrease in the dissociations constants over time could then be reliably explained by an exponentially decaying curve, which at equilibrium, resulted in a Kd of 12 ± 2 µM as the true affinity between lipid-associated PASb-3α and PASb-1β (red solid line). The observed dependence of the binding affinity on time could, in principle, be attributed to both the heterogeneous mixture and the large size of the bacterial lipids (for instance, the phospholipids) bound to the protein. In order to test for these variables, purified PASb-3α was incubated overnight in the presence of a 10-fold molar excess of free linoleic acid and the exchanged excess lipids were removed by dialysis. As shown in the rightmost panel of **(a)**, despite showing a similar behaviour to that described above (with ill-defined dose-response curves for short incubation times) the interaction stabilized already by 20 hours, yielding a similar Kd at equilibrium of 10 ± 1 µM. This observation suggests that a more homogeneous lipid population, coupled with the absence of the glycerol group and larger radicals allowed for faster complex stabilization. **(c)** Intrigued by the identification of such dissimilar behaviour in the dimerization kinetics of PASb-3α1 and PASb-1α with PASb-1β, we set out to test, by NMR spectroscopy, whether distinct interfaces, as well as conformational changes, would be required upon dimerization. For such, PASb-3α and PASb-1α were individually titrated over 15N-labelled PASb-1β, while monitoring the chemical shift and line width changes in the 15N-HSQC spectra. **(d)** Peaks corresponding to residues at C-terminal region of PASb-1β (highlighted in orange) are those which have equivalently undergone the most perturbation upon binding to both PASb-3α or PASb-1α, based on a chemical shift cut-off criteria of Δδ (15N+1H) ≥ 0.04 ppm and relative peak intensity changes equal or greater than -0.4 (in both cases, one standard deviation above average). Peaks corresponding to PASb-1β residues Phe363, Val376, Arg379, Gln421 and Thr462 (labelled in blue) have responded specifically to the presence of PASb-1α, whereas Arg366, His367 and Ile458 (labelled in red) only responded to PASb-3α binding. **(e)** The PASb-1β residues identified by NMR were mapped onto the previously published crystal structure of the PASb-1α:PASb-1β dimer (PDB 4h6j). The PASb-1β interfaces involved in the interaction with these two α isoforms are equivalent within the C-terminal region (orange surface patches), but remarkably different within the N-terminal region (blue surface patches for PASb-1α and red surface patches for PASb-3α). The N-terminal of PASb-1β interacts directly with the N-terminal of the α subunits and there is a displacement of this region of PASb-3α compared to PASb-1α caused by the presence of the lipid in the former, resulting in a steric hindrance in the predicted model of interaction between PASb-3α and PASb-1β. Therefore we conclude that conformational changes may also be necessary for PASb-3α:PASb-1β interaction. Figure drawn by ALBA.

Supplementary Figure 4.

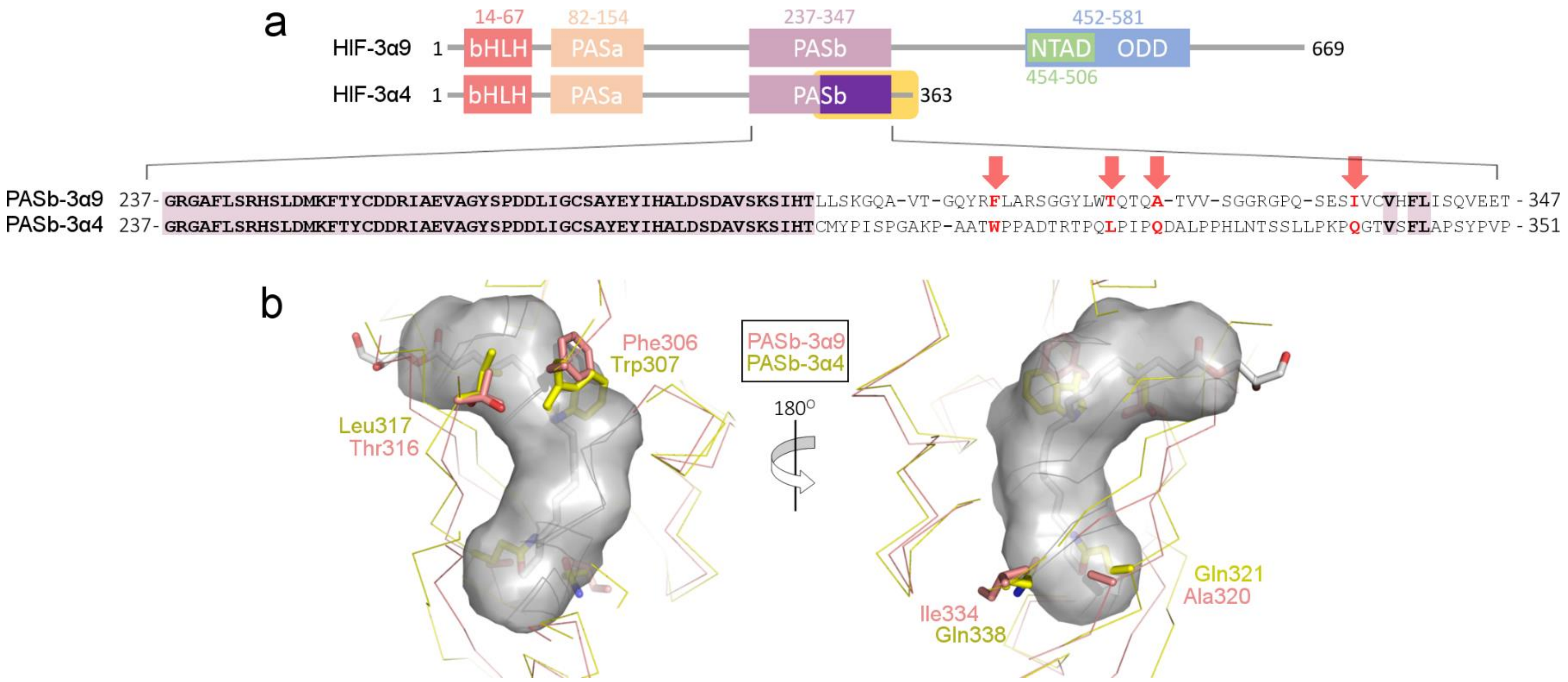

HIF-3α isoform 4 is predicted to be insensitive to fatty acid-derived lipids. (a) The splicing event that generates HIF-3α4 results in fully distinct amino acid sequence for the second half of its PASb domain (purple region, highlighted in yellow) when compared to PASb of all other HIF-3α isoforms. The numbering system used in the crystal structure is in accordance to HIF-3α9, the canonical isoform (Uniprot Q9Y2N7-1). **(b)** Based on the predicted structure of PASb-3α4, by *ab initio* homology modelling, key amino acid substitutions such as Phe306 by Trp307 (in HIF-3α9 and HIF-3α4, respectively), Thr316 by Leu317, Ala320 by Gln321 and Ile334 by Gln338 (indicated by red arrows) may result in occlusion and mischaracterization of the hydrophobic pocket as experimentally determined in PASb-3α (the crystal structure), therefore becoming incapable of binding to fatty acids. Figure drawn by ALBA.

Supplementary Figure 5.

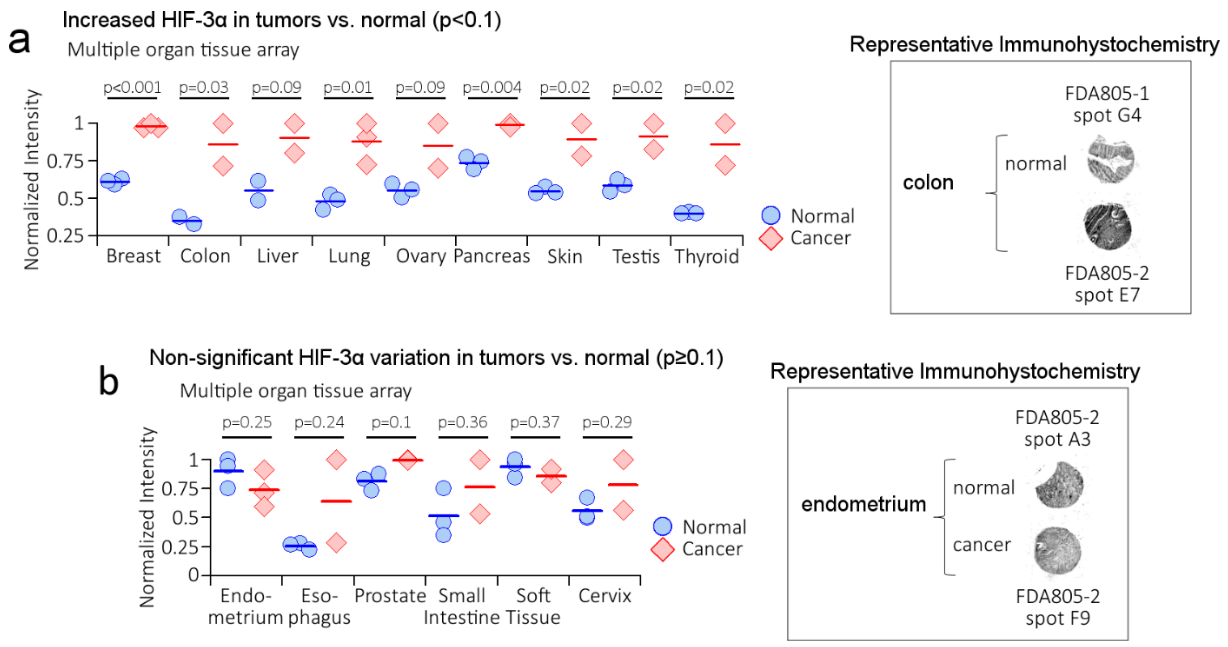

HIF-3α has enhanced protein expression levels, compared to normal tissue counterparts. HIF-3α endogenous expression in tumor cells has been scarcely evaluated to date. Interested as to whether HIF-3α have a physiological role in tumors, we first asked if it was present on a normal vs. cancer tissue array of different types of organs. It can be seen that **(a)** HIF-3α express at higher level on lung, breast, skin, thyroid and abdominal region (colon, liver, ovary, pancreas and testis) tumors in comparison to normal tissue ($p < 0.1$). **(b)** Tissue samples which no statistically differential expression levels of HIF-3α was detected, in tumors when compared to normal tissue, yielding $p \geq 0.1$. Lines indicate the mean. The statistical significance was assessed by a simple unpaired two-sample t-test for the normal versus cancer samples for a given tissue.

Supplementary Figure 6.

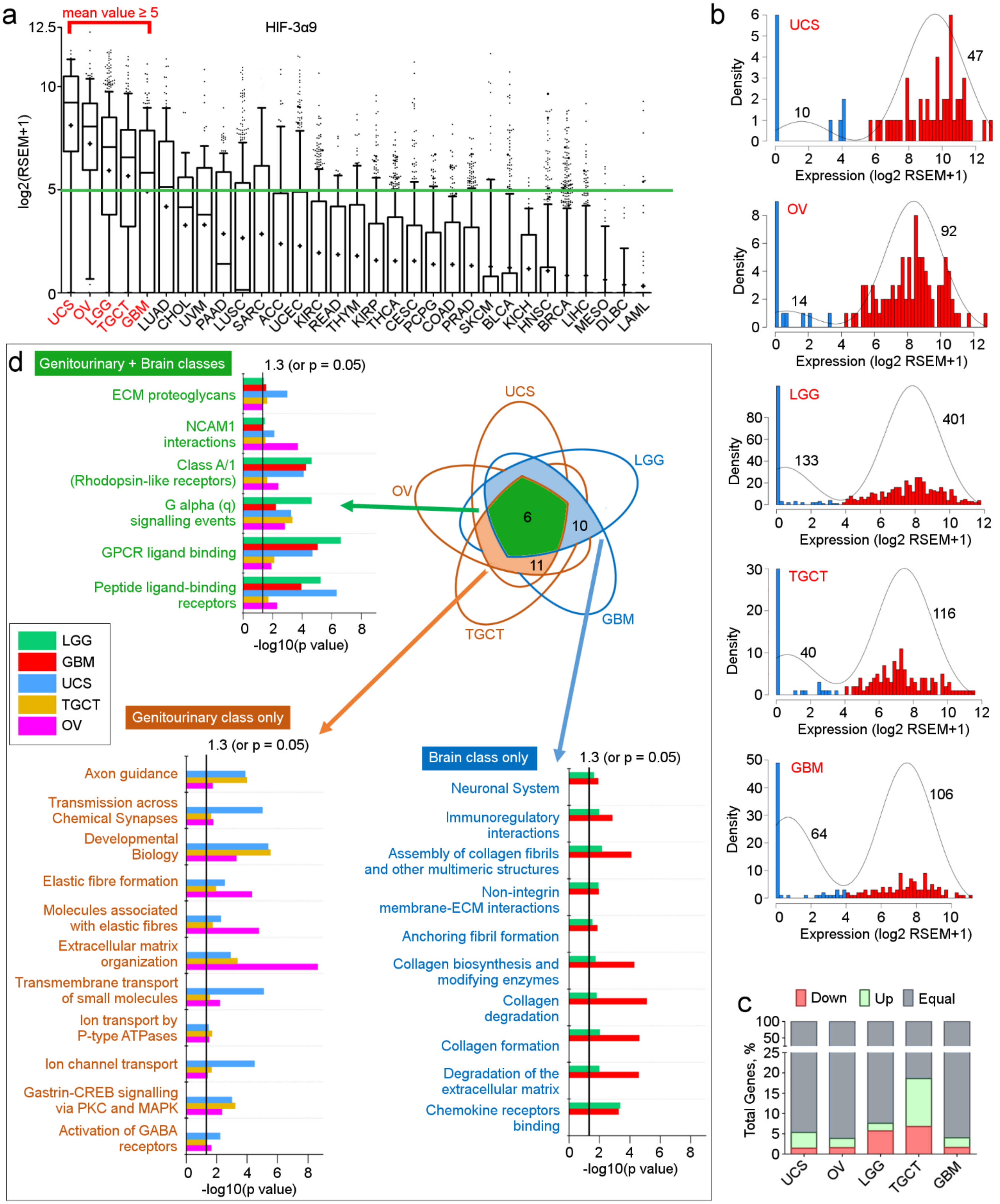

HIF-3α9 mRNA level as detected by RNAseq of several TCGA data available from the Cancer Genome Atlas data portal. Intrigued as to which types of tumors present the highest level of HIF-3α9 expression (the canonical isoform, Uniprot Q9Y2N7-1), we compared its log2(RSEM + 1) values amongst UCS (Uterine Carcinosarcoma), OV (Ovarian serous cystadenocarcinoma), LGG, (Brain Lower Grade Glioma), TGCT (Testicular Germ Cell Tumors), GBM (Glioblastoma multiforme), LUAD (Lung adenocarcinoma), CHOL (Cholangiocarcinoma), UVM (Uveal Melanoma), PAAD (Pancreatic

adenocarcinoma), LUSC (Lung squamous cell carcinoma), SARC (Sarcoma), ACC (Adrenocortical carcinoma), UCEC (Uterine Corpus Endometrial Carcinoma), KIRC (Kidney renal clear cell carcinoma), READ (Rectum adenocarcinoma), THYM (Thymoma), KIRP (Kidney renal papillary cell carcinoma), THCA (Thyroid carcinoma), CESC (Cervical squamous cell carcinoma and endocervical adenocarcinoma), PCPG (Pheochromocytoma and Paraganglioma), COAD (Colon adenocarcinoma), PRAD (Prostate adenocarcinoma), SKCM (Skin Cutaneous Melanoma), BLCA (Bladder Urothelial Carcinoma), KICH (Kidney Chromophobe), HNSC (Head and Neck squamous cell carcinoma), BRCA (Breast invasive carcinoma), LIHC (Liver hepatocellular carcinoma), MESO (Mesothelioma), DLBC (Lymphoid Neoplasm Diffuse Large B-cell Lymphoma) and LAML (Acute Myeloid Leukemia). The upper-quartile normalized isoform-level expression profile of HIF-3α9 (uc002peh.2), whiskers representing the 10- and 90-percentile, respectively, showed the HIF-3α9 level varies amongst the compared types of tumors **(a)**. An established threshold of 5 (green line) indicated 5 types of tumors (all falling within two classes, genitourinary and brain tumors) with the highest expression level. **(b)** To find out genes differentially expressed on the selected tumor types when HIF-3α9 is at its higher expression level, a 2-component Gaussian-mixture distribution model was applied to define samples positive (red) and negative (blue) for HIF3α9. The numbers above the lines indicates the amount of patients in each class. **(c)** The percentage of differentially expressed (up- or down- regulated) genes above a cutoff of 0.95 posterior probability was defined for the selected tumors. **(d)** The REACTOME enriched pathways (using both UP and DOWN differentially expressed genes) bellow p-value 0.05 for the 5 datasets showed that the enriched pathways directly correlated with the ECM proteoglycans, NCAM1 interactions, Class A/1 (Rhodopsin-like receptors), G alpha (q) signaling events, GPCR ligand binding. Nevertheless, the common pathways in genitourinary class include several extracellular matrix remodeling related processes (elastic fiber formation, molecules associated with elastic fibers, extracellular matrix organization), together with signaling pathways (gastrin-CREB signaling via PKC and MAPK) and alterations on general transmembrane transport (transmembrane transport of small molecules, ion transport by P-type ATPases and ion channel transport). As for the nervous-system associated tumors, extracellular matrix related process are also significantly altered (assembly of collagen fibrils and other multimeric structures, non-integrin membrane-ECM interactions, anchoring fibril formation, collagen biosynthesis and modifying enzymes, collagen degradation, collagen formation and degradation of extracellular matrix), as well as immunoregulatory interactions and chemokine receptors binding. The existence of intersecting pathways in two unrelated tissue background groups also indicates a more general importance of the isoform, not limited to the expression context, but with general effects over the biology of the cell. Moreover, both intersecting and non-intersecting REACTOME pathway enrichment points toward a cause-or-consequence relationship between HIF-3α9 expression and an important tumor feature such as ECM remodeling. The potential role of HIF-3α9 on ECM remodeling and thus cell migration and metastasis is worth of further investigation.
